# Drift and ownership toward a distant virtual body

**DOI:** 10.3389/fnhum.2013.00908

**Published:** 2013-12-25

**Authors:** Ausiàs Pomés, Mel Slater

**Affiliations:** ^1^Event Lab, Faculty of Psychology, University of BarcelonaBarcelona, Spain; ^2^Institució Catalana de Recerca i Estudis AvançatsBarcelona, Spain; ^3^Institute for Brain, Cognition and Behaviour, University of BarcelonaBarcelona, Spain

**Keywords:** body ownership illusion, rubber hand illusion, virtual reality, first person perspective, third person perspective, out-of-body experience

## Abstract

In body ownership illusions participants feel that a mannequin or virtual body (VB) is their own. Earlier results suggest that body ownership over a body seen from behind in extra personal space is possible when the surrogate body is visually stroked and tapped on its back, while spatially and temporal synchronous tactile stimulation is applied to the participant's back. This result has been disputed with the claim that the results can be explained by self-recognition rather than somatic body ownership. We carried out an experiment with 30 participants in a between-groups design. They all saw the back of a VB 1.2 m in front, that moved in real-time determined by upper body motion capture. All felt tactile stimulation on their back, and for 15 of them this was spatially and temporally synchronous with stimulation that they saw on the back of the VB, but asynchronous for the other 15. After 3 min a revolving fan above the VB descended and stopped at the position of the VB neck. A questionnaire assessed referral of touch to the VB, body ownership, the illusion of drifting forwards toward the VB, and the VB drifting backwards. Heart rate deceleration (HRD) and the amount of head movement during the threat period were used to assess the response to the threat from the fan. Results showed that although referral of touch was significantly greater in the synchronous condition than the asynchronous, there were no other differences between the conditions. However, a further multivariate analysis revealed that in the visuotactile synchronous condition HRD and head movement increased with the illusion of forward drift and decreased with backwards drift. Body ownership contributed positively to these drift sensations. Our conclusion is that the setup results in a contradiction—somatic feelings associated with a distant body—that the brain attempts to resolve by generating drift illusions that would make the two bodies coincide.

## Introduction

Is it possible to feel that a body you see in extra personal space is actually your body? The answer is obviously ‘yes’ - for it could be a mirror reflection, or a live video stream of your actual body. Suppose though that it is another body that is not in a mirror or a video, but a life-sized, three dimensional body that can be seen in your spatial surroundings a few meters away from your viewpoint: under what circumstances could this be felt to be your body? This issue has received attention in the cognitive neuroscience literature on body representation, concerned with how the brain represents the body. There are two different views—one suggesting that body ownership over a distant body is possible, and another that it is not. In this paper we describe an experiment that provides a unifying interpretation of the disparate results in this area, suggesting that both views are supportable.

Inspired by the multisensory technique used in the rubber hand illusion (Botvinick and Cohen, 1998; Lenggenhager et al., 2007) produced in participants the illusion that a body they were seeing in front of themselves was their body. Participants wore a head-mounted display (HMD) that displayed a video stream from a camera behind them. Hence they saw their own body, facing away from them, through the HMD, and a few meters in front of their own body. An experimenter tapped on their back, and hence they would see the back of the body located in front being tapped while feeling the tapping synchronously on their own back. In an asynchronous condition a delay was introduced so that the visual tapping was not synchronous with the felt tapping. In the synchronous condition participants reported a significantly greater referral of touch than in the asynchronous condition—i.e., they felt the touch as if it were from the body in front—and the touch they felt was being caused by the stick stroking the back of the body in front. Moreover, participants reported a significantly greater feeling that the body they saw was their body in the synchronous compared to the asynchronous condition. When participants in the synchronous condition were blindfolded and moved away from their starting position and asked to walk back to it, there was a significant drift toward the position of the body in front compared to their starting position, but no significant drift in the case of the asynchronous condition. When the body in front was a video of a mannequin body rather than the participant's own body, the illusion (of referral of touch and body ownership) nevertheless occurred and was significantly different between synchronous and asynchronous conditions, and the same result was found for the drift. However, the illusion did not occur when the mannequin was replaced by a wooden block. The seen body had to be of humanoid form.

In a similar but subtly different experiment (Ehrsson, 2007) the subjects wore a pair of HMD s each fed by a video camera from behind, so that they saw a stereo view of their body in front of themselves. However, this time the experimenter tapped synchronously or asynchronously on the chest of the subject and underneath the camera position. Hence the subject would see the stick striking below their visual egocentric position (i.e., the position of the cameras), and feel the stick striking their chest. When the visual and tactile striking were synchronous, participants felt that they were behind their real body—integration between the visual and tactile stimulation shifted their sense of self-location to the position of the camera. This did not occur to the same extent when the visual and tactile stimulation was asynchronous. Moreover, when a hammer was used to apparently attack the space under the camera (the space corresponding to where the participant's body would have been had it coincided with their visual ego-center) then there was significantly greater arousal in the synchronous compared to the asynchronous condition. A further study (Guterstam and Ehrsson, 2012) demonstrated that subjects disowned their real body in the synchronous condition of this illusion.

An experimental setup that combined these two paradigms was presented in Lenggenhager et al. (2009). Subjects laid face down on a bench, with the video camera above them, which fed the HMD. Now subjects would therefore always see their own back, but this time below their visual viewpoint. Tapping on their back (condition *Back*) was seen on the back of the body image in front. Alternatively there was tapping on the chest (*Chest*) producing tactile stimulation while there was visual-only tapping at the place near the camera corresponding to the location of the hypothesized chest seen from that viewpoint. When the tapping was synchronous then in condition *Back* subjects would virtually have moved down toward the body below, while *Chest* subjects would have the illusion of being above the location of their real body (i.e., moving up to the position of the camera). In *Back* subjects tended to affirm the statement that the body in front was theirs, but not so in *Chest*. Subjects had to imagine dropping a ball, and indicate the moment that they thought it would reach the floor. In the *Back* condition the imagined time to reach the floor was less than in the *Chest* condition.

The results of that paper suggest therefore that the critical element is where the visual tapping is seen (and correspondingly felt—the back or the chest). If it seen on the back of the body in front, and integrated through synchrony with the felt tapping, then an illusion of ownership over the body in front can occur, together with a drift toward that body. On the other hand when the visual tapping is seen at the position of the camera behind (and felt on the chest), then there is disownership of the body in front, and the sense of self-location is toward the position of the camera. The visual location of the tapping therefore plays a critical role—provided that the tactile stimulation is synchronous; it integrates the tactile with the visual tapping location to produce a congruent illusion that the body is where the tapping is seen.

However, how can it make sense at all to have an illusion of body ownership over a body that is not even in the visual frame of reference determined by the local coordinate system of the eyes, i.e., when seen from third person perspective (3PP)? In Petkova et al. (2011) it was argued that in the *Back* setup (avatar tapping seen on the back) there is no somatic illusion of body ownership over the distant body, but that the results can be explained through self recognition (indeed even the mannequin wore clothing similar to that of the participant). They claim that first person perspective (1PP) with respect to the surrogate body is an essential aspect of a somatic full body ownership illusion. Some evidence for this is provided in Slater et al. (2010), Maselli and Slater (2013) where, as in Petkova et al. (2011) there were direct comparisons between 1PP and 3PP, with only 1PP associated with the illusion.

In this paper we replicate a version of the original experiment described in Lenggenhager et al. (2007) except that we use immersive virtual reality so that the body in front is a life-sized avatar seen through a head-tracked stereo HMD. Instead of measuring drift directly we measure physiological and behavioral responses of participants to a threat to the body in front. We did not find a significant difference in body ownership between the synchronous and asynchronous conditions. Nevertheless our results do support (Lenggenhager et al., 2007) and because of the different type of measures that we recorded our explanation is also compatible with Petkova et al. (2011). We found this result by going beyond simple and conventional statistical analysis, to look at the data using path analysis which supports the simultaneous evaluation of multiple linear relationships, see, e.g., Kaplan (2009).

## Materials and methods

### Experimental design

The experimental design was single binary factor between-groups with 30 male participants, each assigned to one of the two groups according to a pre-computed pseudo-random sequence^1^, realizing 15 per group.

**Figure 1:**
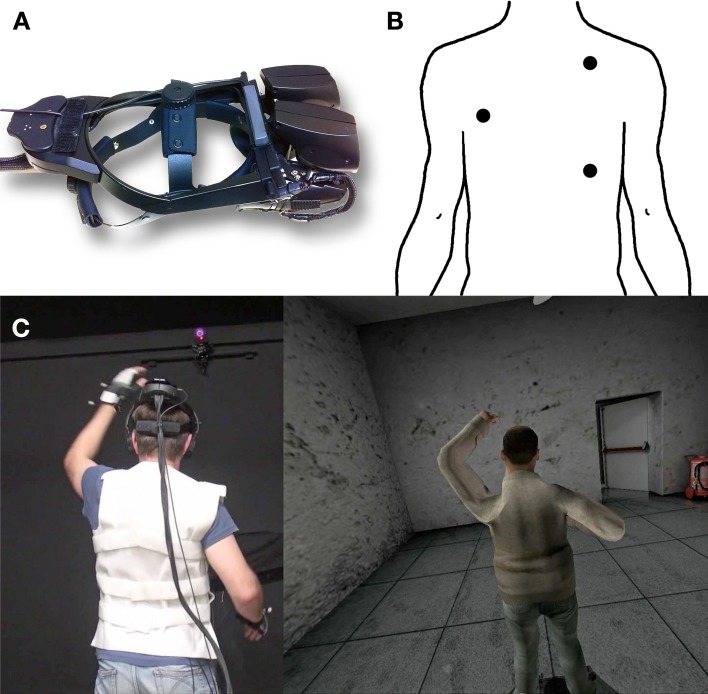
**(A)** Head-mounted display NVIS nVision SX111. **(B)** Predefined points on the back of participants where actuators where placed to deliver vibrotactile feedback. **(C)** The skeleton joints position and rotation where updated through HALCA to follow the upper body configuration of participants.

All participants saw in virtual reality the full body of a male human character (or avatar) 1.2 m in front of their own position. The character was facing away from the participant (Figure 1C). A virtual ball tapped the back of the avatar, while there was tactile stimulation on the back of the participant. The experimental factor was synchrony (Sync) of visual and tactile stimulation on the back of the participant and the back of the avatar, or visuotactile asynchrony (Async). We used a between group design because of the likelihood of strong order effects that make a repeated measures design inappropriate—irrespective of counter balancing. This issue is discussed in more depth in Llobera et al. (2013). We used males only to avoid gender as a possibly confounding factor, and also for a practical reason that the vibrotactile vest used (see 2.2 below) could not be easily fitted to most female bodies. The experiment was approved by Comissió de Bioètica de la Universitat de Barcelona^2^ and the experiment was carried out with written informed consent. Participants were paid 10€ at the end of the experiment. Supplementary Movie 1 illustrates the main parts of the experiment.

The two groups were comparable in their background. The mean ± S.E. age was 30 ± 1.7 in the Sync group and 28 ± 1.2 in the Async group. Based on a pre-experiment questionnaire there were nowhere near significant differences in their level of background computer knowledge, computer programming experience, prior experience of virtual reality, and computer game playing. In particular their prior experience of virtual reality was low (median 2, 75^th^ percentile 3.7 on a scale of 1–7 with 1 meaning no experience and 7 great experience).

### Equipment and software

There were four classes of equipment used: a HMD, head and body-tracking sensors, a haptic vest to automatically deliver vibrotactile stimulation, and physiological recording equipment.

The HMD was an NVIS nVision SX111 (Figure 1A). This displays a 3D scene in stereo with a horizontal field of view of 102° and vertical field of view of 64° by sending left-eye and right-eye images to left and right hand display screens. Its weight is 1.3 kg. Its display refresh rate is 60 Hz. An InterSense IS900 Tracker was mounted on top for head-tracking with 6° of freedom (translation and orientation).

An OptiTrack system with 12 FLEX:V100R2 cameras was used for real-time motion capture of the hand movements of participants. One OptiTrack marker was affixed to each wrist. The tracked information about the arm movements and head movements of the participant was used to animate the upper body of the avatar. The tracking data from the InterSense was used to update the position and rotation of a virtual camera located at the point of view of the participant in the virtual world. The same data was used to update the position and orientation of the head of the avatar, and the configuration of its spine. The data from OptiTrack provided the location of the participant's hands in the physical space, allowing us to position the virtual hands of the avatar accordingly. The position of the elbows of the avatar was computed using inverse kinematics. By combining the tracking data generated from both systems the avatar had approximate but full upper body movements that followed in real-time those of the participant.

The 3D environment was modeled in 3D Studio Max and the experiment was executed in real-time in the XVR framework (Tecchia et al., 2010). The avatar was from a library developed by AXYZ design^3^. The skeleton of the avatar allowed deformation of its underlying meshes through a skinning technique (Mohr et al., 2003). The skeleton configuration (i.e., joints position and rotation) was controlled programmatically within XVR using the HALCA library (Gillies and Spanlang, 2010) (Figure 1, Supplementary Movie 1).

ECG data was recorded at 1024 Hz using a Nexus-4 device with a 3-electrode configuration. Electrodes were placed on the left and right collar bones and the lowest left rib in order to record ECG. ECG data was streamed to a separate file for each participant. Software from g.tec was used to identify the QRS complexes that were also manually inspected and corrected where necessary. From this the heart rate and thereby the heart rate deceleration around the time of a specific threat to the avatar body could be computed.

Haptic feedback was automatically provided by an in-house built Velcro vest with 10 vibrotactile actuators connected to Arduino components. This delivered vibrotactile stimulation to 3 points on the back of the participant, namely the areas around the right middle latissimus dorsi, the left upper latissimus dorsi and the right middle trapezius (Figure 1B). The program caused the stimulation to be in synchrony or asynchrony with corresponding visual stimulation seen on the back of the avatar. Tactile feedback was triggered automatically from the XVR application. A specific library that was developed for this project worked as the tapping controller and was responsible for both the visual feedback (the virtual tapping), and the communication with the device driver that controls the actuators on the vest. The actuators delivered tactile feedback by rotating at 9000 rpm.

All participants experienced tapping for 3 min on their back. The tapping was delivered following a sequence of three points. A tapping sequence is composed of a collection of tapping steps. A *tapping step* is a pair of points in the 3D space that define the origin and the destination of the tapping movement. In our study, the destination is always placed on the surface of the avatar's body and is mapped to the corresponding area of the participant's body.

During the experiment, an object performed the tapping in the virtual environment following a predefined sequence of tapping steps. The way the tapping was applied while following the sequence was determined on the fly. Following previous studies where tapping was delivered by human experimenters, we designed our tapping to produce a similar experience for participants. To move away from a mechanical and predictable visuotactile experience, our system randomizes the main visual and tactile tapping parameters, namely the location and the speed, within some constraints. The tapping follows a predefined sequence of steps, which define the areas where the tapping can be applied. Our system maintains an internal counter to keep track of the current tapping step. We advance this counter based on predefined time increments. Regardless of the fixed tapping sequence, when tapping happens on the *ith* step, our system will randomly move the tapping action between the current *ith* step and steps *i* + 1 and *i* −1. During the experiment we increased the counter every 60 s so that every tapping point would have approximately the same amount of tapping. Regarding the speed, the tapping object moved with a period that was randomized with a minimum of 400 ms and a maximum of 1600 ms.

In the synchronous condition, the tapping system provided congruent sensory input to participants by triggering the vibrotactile actuator in the vest that corresponded to the body part actually receiving the tapping. In this arrangement the actuator was activated when the tapping object reached the destination point of the tapping step. In the synchronous condition the visual tapping that participants saw in the virtual environment matched the tactile feedback from the vest both in place and time. On the other hand, in the asynchronous condition, tactile feedback was delivered by triggering any random actuator and always when the tapping object was not in contact with the virtual body (VB) being tapped (the tapping object was away from the destination point). Actuators were turned on 100 ms after there was visual contact between the tapping object and the surface receiving tapping, and for a random duration between 150 and 350 ms. Random values used in our system were obtained using the *Rand()* function available in XVR's API.

### Procedures

On arrival at the laboratory participants completed a pre-questionnaire that recorded basic information (age, virtual reality experience etc.). Participants also read an information sheet about the experiment and were invited to complete the informed consent form.

Then the experimenter helped participants to place the ECG electrodes and put on the actuated haptic vest and make sure that it fitted tightly to the torso. The experimenter tested that both the Nexus-4 device to measure ECG and the haptic vest were able to communicate with the XVR application as expected. Also, tests were carried out in order to make sure that participants could sense the actuators from the vest.

The experimenter briefed participants with precise instructions about the duration of the experiment, available affordances in the VR system, range of movements and how to act during the several stages of the VR experience. No information was given in advance regarding the actual content of the virtual environment. Participants were encouraged to ask any questions they had about the experiment before proceeding. They were also reminded that they could withdraw from the experiment at any time without giving any reasons.

After this briefing, the experimenter attached two OptiTrack tracking devices to participants' wrists to register the arm movement. The participant then donned the HMD, which was fitted with the InterSense tracker, and adjustments were made to the HMD to ensure a correct stereoscopic view. Finally, they put on a pair of headphones so that they would hear some soft music with the purpose of avoiding distractions from external noises during the experiment.

Just before starting the experiment, the XVR application required a calibration step to correctly register the relationship between the virtual head and arms and the participant's body. Then the experiment started, and participants were asked to look around to become familiar with the virtual environment and to move their upper body at will (namely head, arms, and torso). After 3 min of this, a small virtual ball appeared in the participants' field of view and tapped the avatar body on its back. During that time, subjects were told to remain still and focus on the ball movements. The tapping continued for 3 min.

Then participants experienced an unexpected event. There was a spinning fan in the room and it descended progressively (over 10 s) from the ceiling to the neck level of the avatar standing in front of the participants' point of view in the virtual environment. This followed a similar paradigm to Gonzalez-Franco et al. (2010).

After completion of the experiment they answered a post-questionnaire. Finally, participants were paid 10€ for their time.

### Response variables—questionnaire

The post-questionnaire consisted of 9 questions shown in Table 1, and was adapted from the rubber hand illusion questionnaire (Botvinick and Cohen, 1998) and was similar to that used in Lenggenhager et al. (2007). Q1 and Q2 concerned referral of touch—that the touch was felt on the VB, and that it was caused by the ball. Q3 referred to the illusion of body ownership. It would be expected that there should be high scores in the synchronous condition on each of these three questions, and significantly lower scores in the asynchronous condition. Q4–Q8 in the context of the rubber hand illusion are usually considered as control questions, where low average or high variance scores should be expected, with no difference between the synchronous and asynchronous conditions. Q9 specifically refers to the threat event, and was to assess how much this was taken as a threat to the self.

**Table 1:** **Questionnaire results**.

		**Median ± IQR**		
**Question**	**Label**	**Synchronous**	**Asynchronous**	***P***
Q1 It seemed as if I were feeling the touch of the ball in the location where I saw the virtual body touched.	Referred touch	5 ± 1	2 ± 2	<0.00005
Q2 It seemed as though the touch I felt was caused by the ball touching the virtual body.	Caused by ball	4 ± 1	4 ± 2	0.030
Q3 It felt as if the virtual body was my body.	My body	3 ± 1	3 ± 0	0.422
Q4 It felt as if my (real) body was drifting toward the front (toward the virtual body).	Drifting forward	2 ± 2	2 ± 3	0.897
Q5 It seemed as if I might have had more than one body.	More than one body	2 ± 2	2 ± 2	0.880
Q6 It seemed as if the touch I was feeling came from somewhere between my own body and the virtual body.	Touch between	2 ± 2	3 ± 3	0.366
Q7 It appeared (visually) as if the virtual body was drifting backwards (toward the real body).	Avatar drifting backwards	2 ± 2	1 ± 1	0.180
Q8 During the experiment the body I saw was that of another person.	Another person	3 ± 3	3 ± 2	0.525
Q9 When the fan descended over the head of the virtual body in front, it felt as if it could chop my head.	Head chop	3 ± 3	3 ± 2	0.814

The question scores are on a 1–5 Likert scale with 1 indicating the most disagreement and 5 the most agreement with the statement. The median and interquartile range is given for each question by Synchronous and Asynchronous. The P-value is for test of equality of the two groups using two-sided Wilcoxon rank-sum test (n = 15 in each group).

### Response variables—heart rate deceleration

Heart rate deceleration (HRD) was computed for (i) 6 s before the fan started to descend as a baseline (HRD1) and (ii) for 6 s starting from 2 s before the fan finally stopped. This timing was based on interviews with participants that estimated the moment from when they actually saw the fan descending. HRD has been previously shown to be a response variable that significantly correlates with states of stress and anxiety induced by sudden unpleasant stimuli (Bradley et al., 2001; Cacioppo et al., 2007), and has been successfully used in previous studies as a physiological correlate of the full body ownership illusion (Slater et al., 2010) and other ownership illusions (Tajadura-Jiménez et al., 2012). Since in this experiment the threat was not instantaneous but involved a fan descending toward the head of the body, we could not know the precise moment that participants realized that it would cut the head of the avatar. This was the same situation as in Maselli and Slater (2013), and the method of computing the HRD was as in that paper. To ensure that a sustained deceleration of HR was being captured a time period of at least 0.3 s of decrease in instantaneous HR was required. Additionally, the starting point of this decrease was required to be no more than 2 s from the start of the specific event [i.e., (i) and (ii) above]. HRD was then computed as the ratio [HR(tmax) - HR(tmin)]/(tmin-tmax) where tmin and tmax denote the locations in time of the minimum and maximum HR, respectively. Were the HR monotonically increasing rather than decreasing, then a similar method would produce a heart rate acceleration. If the HR was found not to be monotonically decreasing or increasing in the period under consideration then it would have been recorded as zero (but this did not occur).

### Response variables—head movement distance

Head tracking data was recorded throughout the experiment. This allowed computation of the distance through which the head tracker moved at any time period, by taking the integration over time of the successive tracking positions. We computed the distance that the head moved 10 s prior to the onset of the threat event as a baseline (d1), and 10 s during the threat starting from 2 s after the fan started to descend (d2).

### Statistical methods

For analysis of the questionnaire data as response variables we used the non-parametric Wilcoxon rank-sum test to test the difference between the conditions (Sync, Async). To test whether the condition influenced the HRD and distance data we used ANCOVA with d2 and HRD2 as the respective dependent variables, condition (Async = 0, Sync = 1) as the factor and d1 and HRD1 as the covariate. To examine whether the physiological and distance variables related to the questionnaire responses we used regression analysis.

All of the above are single equation techniques, meaning that we are unable to unravel more complex (and realistic) simultaneous multivariate relationships between the variables. For example, a feeling of body ownership might simultaneously influence the physiological response to the threat (HRD2) and the distance that the head moved (d2), but these simultaneously are influenced by how much the participant tends anyway to move the head (d1) and have heart rate decelerations (HRD1). Conventional approaches have to separate these into separate stochastic linear models (whether ANOVA or regression they are all instances of the general linear model) that cannot assess multiple simultaneous effects. For this purpose we turned to path analysis, for example (Kaplan, 2009), where such effects can be appropriately modeled. We have used this method before in the context of body ownership studies (Kilteni et al., 2012, 2013; Llobera et al., 2013). For the path analyses we used Maximum Likelihood estimation, available in Stata 13 (www.stata.com).

## Results

### Questionnaire results

**Figure 2:**
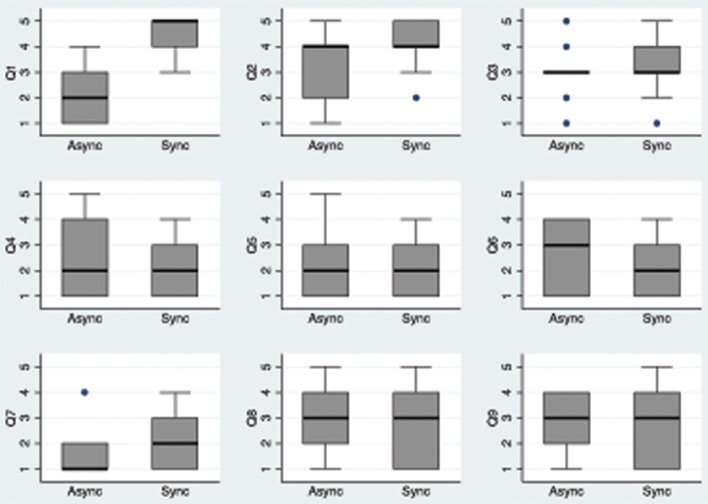
**Box plots of the questionnaire data by condition.** The thick horizontal lines are the medians, the boxes are the interquartile (IQR) ranges, the whiskers extend to the highest or lowest data point within 1.5 ^*^ IQR. Values outside of this range are marked by single points.

The questionnaire results are shown in Table 1 and Figure 2. Q1 indicates a referral of touch toward the avatar body. There is a strong difference between synchronous and asynchronous conditions, as would be expected from earlier results. This also serves to validate the technique used for delivering the tactile stimulation automatically using the vibrotactile vest. Q2 apparently suggests that the scores are similar between synchronous and asynchronous. However, the difference between these two is significant, and the reason for this is shown in the box plot (Figure 2).

There is no evidence for strong body ownership (Q3), both median scores are at the mid-point of the scale and there is no significant difference between them, however the distributions of scores are not the same with the majority of scores at the mid-point of 3 in the Async condition (Figure 2).

The control questions Q4–Q8 are as would be expected—low median scores with no significant difference between the Sync and Async conditions. Finally the same is true for Q9.

### Head movement distance and HRD

Table 2 shows the means and standard errors of the head distance travelled before and after the threat, and for the Async and Sync conditions. There is an overall greater average head movement as a result of the threat compared to immediately before it. There appears to be a greater impact in the Async condition on head movement. However, ANCOVA of d2 on the condition (Async, Sync) with d1 as a covariate shows no significance effect of condition [for the interaction term *F*_(1, 26)_ = 0.00, *P* > 0.98], and for the main effect without the interaction term *F*_(1, 27)_ = 2.28, *P* >0.14). So although the descending fan had an effect on head movement this does not seem to be affected by the condition.

**Table 2:** **HRD and Distance Results**.

		**Before**		**After**		***P* (paired *t*-test, 2 tailed)**
		**Mean**	***SE***	**Mean**	***SE***	
	**Condition**					
Distance (m)	Async	0.27	0.050	0.40	0.077	
	Sync	0.25	0.044	0.29	0.050	
	Combined	0.26	0.033	0.34	0.046	0.010
HRD	Async	1.32	0.540	2.67	0.655	
	Sync	0.93	0.709	1.58	0.952	
	Combined	1.12	0.439	2.13	0.577	0.037

Means and Standard Errors of Head Movement Distance and HRD by Condition (Async, Sync) and Before and After the Threat

Table 2 shows similar results for the HRD. Again there is a greater overall average HRD after the threat event compared to before, but apparently no effect of condition. The interaction term in the ANCOVA has *F*_(1, 26)_ = 1.33, P > 0.25, and the main effect without including the interaction has *F*_(1, 27)_ = 0.72, P > 0.40.

### Discussion of initial results

Considering the results so far we find little support for the conclusions reached in Lenggenhager et al. (2007). Although there is evidence for referral of touch, there is no evidence of the effect of the synchronous condition on body ownership, and although there are physiological and behavioral correlates of the threat to the avatar these do not vary by condition.

This conclusion moreover is not too out of line with that of Lenggenhager et al. (2007) with respect to the body ownership (Q3). In Figure 2B of that paper (p1098) the mean score for Q3 appears to be about 1 in a range of scores from −3 to 3. This equates to about 3.6 on our scale of 1–5. The mean (rather than the median) of Q3 in our experiment in the synchronous condition is almost the same, 3.3. However, the critical difference between the two results is that in Lenggenhager et al. (2007) there is a significant difference between Sync and Async whereas in our experiment there is apparently not.

The other issue in relation to Lenggenhager et al. (2007) is that drift was measured with respect to blindly walking toward the body in front after a displacement, rather than our approach of measuring physiological and behavioral responses to a threat to the body. However, we can examine a subjective counterpart of drift from the questionnaire data using Q4 (Drifting Forward) and Q7 (Avatar Drifting Backwards). If the participants felt themselves to be moving toward the avatar in front during the threat then this should put them in danger. However, if they had the illusion that the avatar was drifting back toward their position, then this would put the avatar out of danger. Hence if there are these illusions of drift then we should expect the distance measure and HRD to reflect this—increasing with Q4 and decreasing with Q7. This, to our surprise, is what happened.

We carried out a regression analysis of d2 on d1, condition, Q3, Q4 and Q7 allowing for interactions between condition and the other variables. After removing all non-significant interaction effects and main effects not associated with significant interaction effects, we arrive at the result shown in Table 3. This shows a very strong positive association between Q4 and d2 independently of condition. It also shows a strong negative effect of Q7 but only in the Sync condition.

**Table 3:** **Regression of d2 on d1, condition (Async, Sync) and Q4 (Drifting Forward)**.

	**Coefficient**	***SE***	***t***	***P***	**Partial η^2^**
Constant	−0.19	0.10	−1.79	0.086	
Main Effect: Sync	0.21	0.11	1.81	0.083	0.12
d1	1.14	0.15	7.65	0.000	0.71
Main Effect Q4	0.08	0.02	3.50	0.002	0.34
Main Effect Q7	0.06	0.05	1.27	0.218	0.06
Interaction: Sync.Q7	−0.16	0.06	−2.62	0.015	0.22

Overall F_(5, 24)_ = 15.50, P < 0.00005, R^2^ = 0.76. Shapiro-Wilk test for normality of residual errors has P > 0.62. Sync = 0 is asynchronous and Sync = 1 is synchronous. The interaction term Sync.Q7 = 0 for asynchronous and Q7 for synchronous.

Table 4 shows the results of following the same strategy with HRD, regressing HRD2 on condition including possible interactions with HRD1, Q3, Q4 and Q7. Here Q3 is positively associated with HRD2, and in the synchronous condition only Q4 is positively associated and Q7 negatively associated with HRD2.

**Table 4:** **Regression of HRD2 on HRD1, condition (Async, Sync), Q3 (My Body), Q4 (Drifting Forwards) and Q7 (Avatar Drifting Backwards)**.

	**Coefficient**	***SE***	***t***	***P***	**Partial η^2^**
Constant	−2.28	1.76	−1.30	0.209	
Main Effect: Sync	2.33	1.84	1.27	0.219	0.07
HRD1	0.58	0.19	3.13	0.005	0.31
Main Effect: Q3	1.21	0.50	2.44	0.023	0.21
Main Effect: Q4	−0.05	0.40	−0.12	0.905	0.00
Main Effect: Q7	0.47	0.70	0.67	0.511	0.02
Interaction: Sync.Q4	4.97	1.88	2.65	0.015	0.24
Interaction: Sync.Q7	−7.25	1.91	−3.80	0.001	0.40

Overall F_(7, 22)_ = 6.94, P = 0.0002, R^2^ = 0.69. Shapiro-Wilk test for normality of residual errors has P > 0.45.

Hence overall the evidence suggests that the feeling of moving forward was associated with a greater response to the threat indicated by more head movements and higher HRD, and the feeling that the VB was moving backwards was associated with a diminution of HRD and head distance movement.

### Path analysis

One problem with the above analysis is that there are individual equations for d2 and HRD2, whereas the feelings represented by Q4 and Q7 may simultaneously affect both HRD and head movement. Moreover the role of Q3 (My Body) might be considered not as directly influencing HRD but mediated through its influence on Q4 and Q7. It would be interesting to consider these relationships together in one overall model, for which we turn to path analysis. Path analysis is a special case of Structural Equation Modeling (SEM) restricted to models that include only observed exogenous and endogenous variables, but not latent variables. A path model can be thought of as a set of simultaneous stochastic equations, where a dependent variable of one equation can be an independent variable in another. Based on any model specification (i.e., set of equations) the overall covariance matrix is estimated, typically through maximum likelihood estimation. The standard general linear model (which encompasses both multiple regression, ANOVA and ANCOVA) can be considered as a special case restricted to the situation where there is only one dependent variable (i.e., only one equation). Path analysis was invented in the 1920s (Wright, 1921) and see Kaplan (2009) for a modern treatment.

Path analysis and more generally SEM can be used either for confirmatory analysis (to test a specific model hypothesis) or for exploration. Here we employ path analysis as an exploratory tool, to generate new hypotheses that arose due to the experiment, for testing in later studies.

Our basic path model is based on the idea that the illusion of body ownership (Q3) should drive the illusions of moving forward (Q4) and that the avatar is moving backwards (Q7) (see Discussion). These in turn may be associated with changes in HRD and head distance.

**Figure 3:**
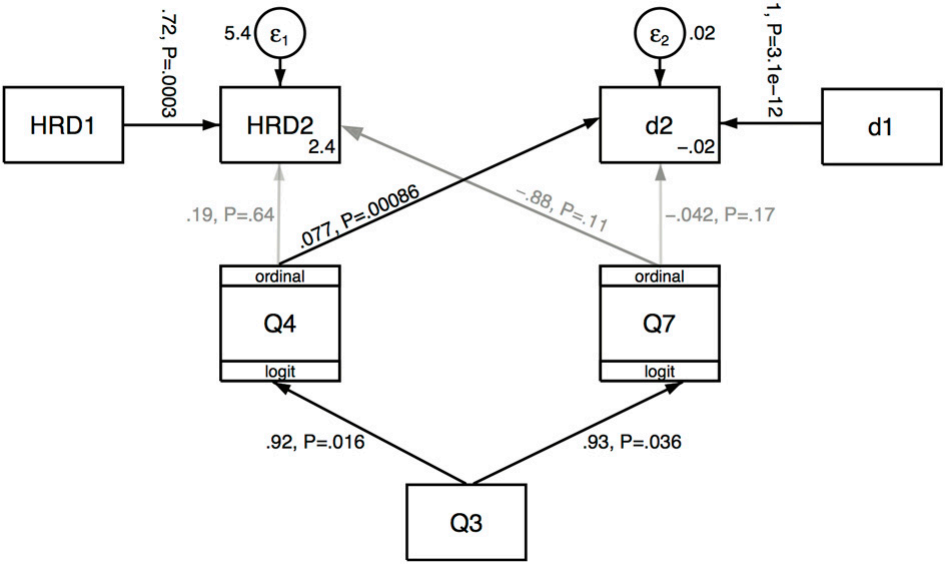
**Path analysis for HRD and distance measures in relation to questionnaire variables Q3, Q4, Q7.** The values on the paths are the path coefficients followed by their significance levels. The epsilon terms represent the random error term. The diagram can be interpreted as a set of simultaneous linear prediction equations, e.g., HRD2 = 2.4 + 0.19^*^Q4 − 0.88^*^Q7 + 0.72^*^HRD1 + epsilon. The interpretation of the paths to the ordinal variables (Q4, Q7) is more complex (see main text). The circles are the random error terms and values by the epsilon circles are their variances. The lighter arrows indicate the non-significant paths.

Figure 3 shows the path diagram that corresponds to this model. The boxes correspond to the variates and the paths correspond to hypothesized directions of causation. There are two types of variate: those indicated by plain boxes with arrows coming in (HRD2 and d2) represent linear models, so that for example the expected value of HRD2 is a linear function of HRD1, Q4 and Q7 (analogous to normal regression). HRD2 and d2 are such endogenous variables—they are determined within the model. However, HRD1, d1 and Q3 are exogenous variables (they are observed values not determined within the model).

Q4 and Q7 are also endogenous variables but of a different type. Since these are ordinal they should not be modeled as continuous random variables, but rather we use an ordered logistic model. Here if *y*_*i*_ is the *i*th observation on an ordered variable with possible values 1,2,…,*q*, and η_*i*_ = ∑^*m*^_*s* = 1_β_*s*_*x*_*is*_ is the *i*th observation on a linear predictor for *m* independent or explanatory variables *x*_1_,… *x*_*m*_ then the ordered logistic model is given by $P(y_{i}\leq j)=1/(1+\exp(-(k_{j}+\eta_{i}))$, where *P*() denotes probability. The *k*_*j*_, *j* = 1, …, *q*, are ‘cut points’ with *k*_*q*_ = ∞. The coefficients β_*s*_ and the cut points are typically estimated by maximum likelihood.

**Figure 4:**
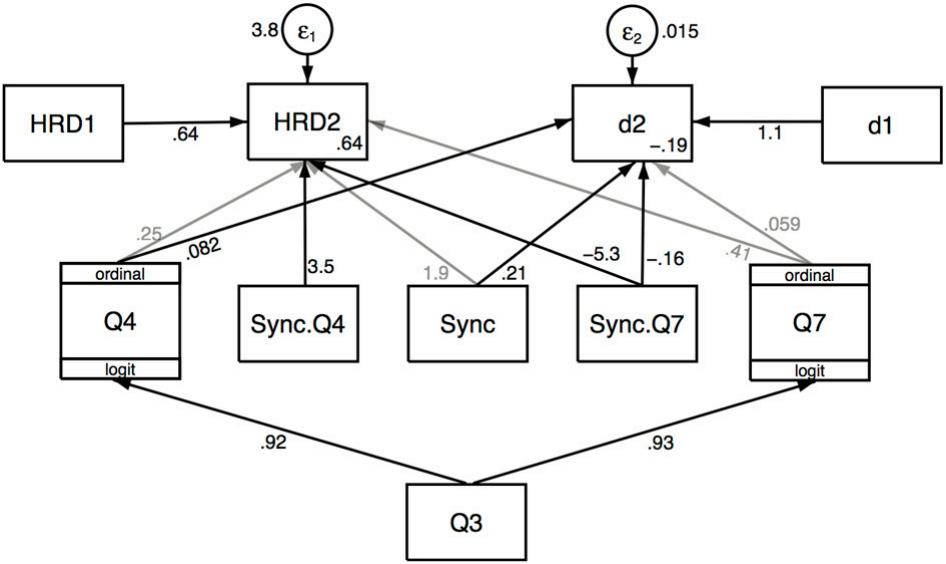
**Path analysis for HRD and distance measures in relation to the synchronous/asynchronous condition, and questionnaire variables Q3, Q4 and Q7.** The values on the paths are the path coefficients. Table 5 gives full details.

Figure 3 shows that this baseline model is not a good one, with no significant effect of Q4 or Q7 on HRD2, although there is a significant positive association between Q4 and d2, and also Q4 and Q7 are positively associated with Q3. However, this model does not take into account the experimental conditions (Async and Sync). Figure 4 shows a model that introduces the condition, including interaction terms. Interaction terms that were not significant have been deleted, and main effects included whether significant or not when the main effect variable is included in a significant interaction effect. A likelihood ratio test of this model compared to the baseline one has χ^2^(5) = 19.08, *P* < 0.002, showing a great improvement in the fit. Moreover the AIC (Akaike Information Criterion) reduces from 282 in the baseline model to 273 in the second model. If further terms are added (for example, a path from Sync to Q3) then there is no further gain in goodness of fit or notable reduction in AIC (but see Discussion). Table 5 presents the equations and parameter estimates in detail. The last column of the table shows that the model fitted values of the endogenous variables correlate well with the true values.

**Table 5:** **Path Analysis Results Corresponding to Figure 4**.

**Terms**	**Coefficient**	***SE***	***z***	***P***	**r, *P***
**HRD2**					0.78, 0.0000
Const.	0.64	1.24	0.51	0.607	
HRD1	0.64	0.18	3.62	0.000	
Sync	1.93	1.76	1.09	0.275	
Q4	0.25	0.37	0.68	0.497	
Q7	0.41	0.67	0.61	0.539	
Sync.Q4	3.54	1.72	2.06	0.040	
Sync.Q7	−5.28	1.67	−3.17	0.002	
**d2**					0.87, 0.0000
Const.	−0.19	0.09	−2.00	0.045	
d1	1.14	0.13	8.56	0.000	
Sync	0.21	0.10	2.02	0.043	
Q4	0.08	0.02	3.91	0.000	
Q7	0.06	0.04	1.42	0.157	
Sync.Q7	−0.16	0.05	−2.93	0.003	
**Q4**					0.45, 0.013
Q3	0.92	0.38	2.41	0.016	
**Q7**					0.44, 0.016
Q3	0.93	0.44	2.10	0.036	

The path analysis uses Maximum Likelihood estimation. For example the fitted equation for d2 is *−0.19* + 1.14^*^d*1* + 0.21^*^ Sync + 0.08^*^Q4 + 0.06^*^Q7−0.16^*^(Sync.Q7). Sync = 0 for asynchronous and Sync = 1 for synchronous. Sync.Q4 is an interaction term which is zero for asynchronous and Q4 for synchronous. P = 0.000 means P < 0.0005. The final column (r, P) shows the Pearson correlation coefficient between the observed and fitted values in the case of HRD2 and d2. For Q4 and Q7 it shows the Spearman correlation between the fitted values of the linear predictor and the observed ordinal values. In each case P is the corresponding significance level for the test that the correlation is 0.

## Discussion

We have carried out a partial replication of the experiment described in Lenggenhager et al. (2007) to examine whether it is possible to reproduce the results of a body ownership illusion toward a VB located in extra personal space, under conditions of synchronous visuotactile stimulation. Using statistical tests individually on a set of questionnaire scores and physiological and behavioral responses to a threat to the VB, we found that the most important earlier results were not replicated. The visuotactile synchrony did result in a referral of touch illusion, where participants felt touch as if it were located at the body in front, and that the touch was caused by a virtual ball striking that body. Here there were significant differences between the visuotactile synchronous and asynchronous conditions, in line with the earlier paper. However, there was apparently no evidence of a body ownership illusion that varied systematically with the visuotactile condition. One could interpret the scores on the body ownership question as random with median equal to the mid-point of the Likert scale, unrelated to the experimental condition. Moreover, there was apparently no difference in response to the threat event between the two conditions.

However, a path analysis revealed results that are compatible with the earlier paper. The most striking is that HRD2 and d2 increase or decrease were associated with the illusion of drifting forwards (toward the danger) or the VB drifting backwards (away from the danger). The first of these can be interpreted as the participant illusorily entering into the danger zone, and the latter the VB moving out of it. The absolute median scores of Q4 (moving toward) and Q7 (avatar moving backwards) are similar to those in Lenggenhager et al. (2007) on the same questions. However, the analysis here has shown a relationship between the physiological response and the illusory drift. Second, the extents of these illusory drifts are positively associated with the degree to which the avatar body is affirmed as the participant's body.

The fact that these results do not clearly show up in the data, but only with a more sophisticated analysis are grounds for caution. In our experiment participants spent 3 min looking around the environment and moving their bodies during which the avatar in front carried out the same movements in synchrony (Supplementary Movie 1). They then spent another 3 min with visuotactile synchrony or asynchrony. Although in this second phase they were asked not to move, any small inadvertent head or upper body movements would have been reflected in movements of the avatar in front. It is possible therefore that the dominant modality was visuomotor synchrony. To our knowledge there have been no published studies on the relative impact of visuotactile compared to visuomotor synchrony on full body ownership illusions, though a recently completed experiment has suggested that visuomotor dominates (Kokkinara and Slater, under revision). This is a possible explanation as to why the visuotactile had no clear effect in producing the illusion of body ownership, since always the synchronous visuomotor component dominated. Accepting this interpretation means that a few minutes of visuomotor synchrony in this setup can wipe out any effect of visuotactile synchrony, which is an interesting result in itself. However, it should be noted that there is not a vast difference between the original setup (Lenggenhager et al., 2007) and our one—because in the original the distant body was produced by real time video of the actual body. Hence any body movements that the participants in that earlier experiment might have happened to make inadvertently would have also been reflected exactly in the distant body. In their second within-groups study participants were exposed to the video of their real body, a mannequin body, and a non-corporeal object (in counter-balanced order). In this case there was a substantial (if not significant) difference between Q3 in the own body case (just over 2, on a scale of −3 to 3) compared to the mannequin (about 0.5) (Supporting Online Material, Figures S2 A,B). Perhaps this difference was due in part also to the fact that the mannequin body could never move.

A second reason for treating the results cautiously is that we carried out our experiment on males only whereas in Lenggenhager et al. (2007) 9 out of their 14 participants were female. To our knowledge there are very few studies that report any attempt to measure gender differences in body ownership illusions. In a study of the mirror box illusion (Egsgaard et al., 2011) found evidence of greater plasticity with respect to this body illusion in men compared to women. In Burrack and Brugger (2005) it was found that in two vibratory illusions and across a number of measures the only significant differences between men and women were a greater vividness of an arm movement illusion for women, and a correlation between a perceptual aberration measure and subjective illusion of nose extension for men but not for women. However, no gender differences were found in a video based version of the rubber hand illusion (Ijsselsteijn et al., 2006). In results from our own laboratory with respect to the full body ownership illusion, no gender differences were found with respect to body ownership ratings in two studies each of which included approximately equal numbers of both genders (Banakou et al., 2013; Maselli and Slater, 2013). Since there have been well over a thousand papers published in the domain of the rubber hand and associated illusions it would have been well-reported by now if there were important gender differences. So although the difference between gender balance of participants in Lenggenhager et al. (2007) and the current study should be taken into account, this is unlikely to be an important limitation of the current study.

The relatively low body ownership score (median 3 out of a maximum score of 5) may be due simply to difficulty in obtaining a sensation of body ownership with respect to a remote body. There is evidence that body ownership illusion does not occur for a body that is in extrapersonal space when it is directly compared to a body that spatially coincides with the own body seen from 1PP. In Slater et al. (2010) a full body ownership illusion was produced in males with respect to a female VB, when there was 1PP with respect to the VB, so that the VB substituted the person's real body as seen through a wide field-of-view head tracked HMD. When the same body was seen from a third person perspective, slightly outside the position of the real body, then the strength of the illusion was significantly lower. In Petkova et al. (2011) there was a similar finding that the full body ownership illusion was produced only with a 1PP view of the VB and not with a third person viewpoint, and this was again replicated in Maselli and Slater (2013). However, in Gonzalez-Franco et al. (2010) a third person viewpoint with respect to a VB did result in a subjective illusion of ownership, and a behavioral response to a descending fan threatening the exterior VB. Here the manipulation was with upper body visuomotor synchrony or asynchrony, and the illusion was obtained only in the synchronous condition. A critical difference though from our current experiment is that the VB was presented as a mirror reflection, rather than seen from the back.

It was suggested in Petkova et al. (2011) that an explanation for indications of a full body ownership when the body is seen from 3PP is that it is a case of self-recognition rather than a somatic illusion of ownership (i.e., that in the case of 3PP the body is recognized as their body by participants but is not associated with the location of where the participant somatically feels to be). But if the body is recognized as the own body, and a threat toward it is perceived, this is likely to result in some reaction—either to avoid the potential harm, or to protect the body. In the case of our experiment this manifested as greater HRD and a greater propensity to move the head during the harm.

However, according to Q1 (Referred Touch) and Q2 (Caused by Ball) there was feeling associated with the distant body. In these circumstances, therefore, the brain has a contradiction to resolve. There is evidence that is leading to the conclusion that your body is ‘over there,’ but also evidence (the visual egocenter) that points to you being ‘here.’ There is moreover a distinction between where you feel yourself to be (‘here’) and where your body might be (‘there’). Ways to resolve this contradiction are either to move yourself to ‘there’ (Q4 Drifting Forward) and/or to move the other body back toward you Q7 (Avatar Drifting Backwards). Each of these potential actions though has consequences in relation to the threat—the first making it more and the second making it less dangerous. Therefore the contradiction in the setup results in illusions of drift that are consequential for responses to the harm that might be caused by the descending fan. These illusions of drift are at least partially driven by the illusion of body ownership (Q3). While it may not be possible to have a somatic illusion of ownership over a distant body the brain attempts to resolve the contradiction through illusions of drift that would make, one way or another, the real and VB coincide. This mechanism does not contradict either standpoints mentioned in the introduction to this paper: there is an attempt by the brain, through drift, to make the distant body ‘owned’. Of course in direct comparisons between 1PP and 3PP the evidence suggests that 1PP results in the stronger illusion, but when the type of 3PP setup is considered by itself, it is still possible for participants to validly affirm a body ownership illusion over the distant body, but this could reflect more a process of attempting to resolve the contradiction between the feelings seemingly associated with the remote body, and the sense of self location at the actual position of the real body.

Table 1 shows that the variables critical to our analysis (Q4 and Q7) have substantial inter-individual differences, which are only partially explained by the model, through variation in Q3. Hence there are likely to be other psychological factors, not included in our model that accounts for this variation, and likewise the variation in Q3.

Moreover, Table 2 shows that overall the threat event had an impact on HRD and the distance measure, since both are greater in the period after the threat than before. However, the path analysis suggests that d2 and HRD2 are only related to Q4 and Q7 in the synchronous condition but not in the asynchronous. However, these two findings are not inconsistent. The first indicates that the threat was effective as a threat (i.e., in both conditions participants responded to the threat). This could be explained even when there is no illusion of body ownership by the response to seeing a third person attacked. The second result indicates that only in the synchronous condition was the response to the threat also related to other subjective responses, so that the visuotactile stimulation had an effect—which we explain above as producing a contradiction caused through feelings of ownership with respect to a distant body.

Considering the relationships between Q4 and Q3, and between Q7 and Q3 by themselves the correlations between them are only significant in the synchronous condition (Q3 and Q4, asynchronous condition: Spearman's ρ = 0.42, *P* = 0.12; synchronous condition: ρ = 0.60, *P* = 0.02; Q3 and Q7, asynchronous condition: ρ = 0.14, *P* = 0.63; synchronous condition: ρ = 0.69, *P* = 0.004). Including condition (Async, Sync) and the interaction term Sync.Q3 in the path analysis as contributing to each of Q4 and Q7, the path from Sync.Q3 significantly and positively contributes to Q7 (*P* = 0.046) with no other changes to the results. Comparing this new model (i.e., with new paths from Sync and Sync.Q3 to Q7) with the previous model shown in Figure 4 the likelihood ratio test indeed shows a small improvement (χ^2^(2) = 6.05, *P* = 0.046). The evidence does suggest therefore that something additional is occurring in the synchronous condition. In that condition the model indicates that the level of ownership drives Q4 and Q7, and that it is these illusions that are contributing to HRD and the head movement distance over and above what might normally occur when seeing a threat to any body. In other words ownership plays a critical role (but clearly not an exclusive one).

The results of this experiment, and the exploratory path analysis, produce quite an interesting set of findings. However, it is important to note that given the limitations of the study discussed above, that these are taken only as hypotheses to be rigorously tested in future work.

## Author contributions

The experiment was conceived by Mel Slater and designed by Mel Slater and Ausiàs Pomés. The implementation and experiment were carried out by Ausiàs Pomés. The analysis of results was carried out by Mel Slater, and the paper written by Mel Slater in conjunction with Ausiàs Pomés.

### Conflict of interest statement

The authors declare that the research was conducted in the absence of any commercial or financial relationships that could be construed as a potential conflict of interest.

## Footnotes

^1^http://www.randomizer.org

^2^http://www.ub.edu/recerca/comissiobioetica.htm

^3^http://www.axyz-design.com
